# Everything, everywhere, almost at once

**DOI:** 10.7554/eLife.95362

**Published:** 2024-01-29

**Authors:** Jacob Kæstel-Hansen, Nikos S Hatzakis

**Affiliations:** 1 Department of Chemistry, Novo Nordisk Foundation Center for Optimized Oligo Escape and Control of Disease, University of Copenhagen Copenhagen Denmark

**Keywords:** proteins, single molecule tracking, high throughput analysis, cells, receptors, estrogene, Human

## Abstract

A new platform that can follow the movement of individual proteins inside millions of cells in a single day will help contribute to existing knowledge of cell biology and identify new therapeutics.

**Related research article** McSwiggen DT, Liu H, Tan R, Puig SA, Akella LB, Berman R, Bretan M, Chen H, Darzacq X, Ford K, Godbey R, Gonzalez E, Hanuka A, Heckert A, Ho JJ, Johnson SL, Kelso R, Klammer A, Krishnamurthy R, Li J, Lin K, Margolin B, McNamara P, Meyer L, Pierce SE, Sule A, Stashko C, Tang Y, Anderson DJ, Beck HP. 2023. A high-throughput platform for single-molecule tracking identifies drug interaction and cellular mechanisms. *eLife* **12**:RP93183. doi: 10.7554/eLife.93183.

Proteins play a role in almost all cellular processes and are essential for maintaining life across species and organisms. This means that their aberrant function is a major cause of disease. If one could look directly inside cells, they would see a seemingly chaotic scene of proteins continuously moving around. The motion of each protein is heterogeneous in time and space, and linked to its role within the cell. It is also heavily influenced by the local cellular environment and interactions with other molecules (Lippincott-Schwartz et al., 2001; Kusumi et al., 2014).

Conventional research techniques average the behavior of a large number of unsynchronized molecules, and thus fail to account for these variable factors, which are essential for understanding the biology of proteins. This is where single-molecule tracking methods come into play (Chenouard et al., 2014 ). Traditional ways for tracking individual molecules rely on advanced fluorescence microscopy, single-particle tracking and super-resolution imaging to directly observe the movement and interactions of proteins (Kusumi et al., 2014; Sahl et al., 2017; Shen et al., 2017).

These approaches provide the required spatiotemporal resolution, but typically can only analyze a few cells under limited conditions, offering a narrow glimpse of the vast and dynamic world of proteins. Although high-throughput microscopy has become much refined, scaling single-particle tracking remains a challenge (Park et al., 2023; Malle et al., 2022). A method that could record the performance of every single protein inside millions of individual cells, as well as thousands of molecular compounds, and analyze how they move, interact and respond to therapeutics, would be a major scientific breakthrough – one that may soon be a reality.

Now, in eLife, Hilary Beck and colleagues at Eikon Therapeutics and University of California Berkeley – including David McSwiggen as first author – report a high-throughput tracking (htSMT) platform that makes it possible to observe and analyze the behavior and movement of single proteins and molecules on an unprecedented scale (McSwiggen et al., 2023). The platform involves a robotic system capable of autonomously handling reagents and collecting sequential microscopy movies that are then computationally processed to obtain the trajectories of individual proteins within cells and even cellular compartments. This system allows users to image over a million cells, track thousands of individual proteins per cell, and screen thousands of compounds in a single day.

McSwiggen et al. then tested the platform on estrogen receptors and investigated how over 5,000 compounds affected their motion, analyzing hundreds of thousands of cells twice in a single day (Figure 1). This revealed a new correlation between the dynamics of estrogen receptors and the ability of their antagonists to suppress the growth of cancer cells, which conventual methods have failed to detect previously. Moreover, the htSMT platform also revealed whether the tested molecules affect estrogen receptors directly or indirectly through other biological targets that are known to modify the receptor. This provides an unprecedented and unbiased analysis of a complex biological pathway.

**Figure 1.**
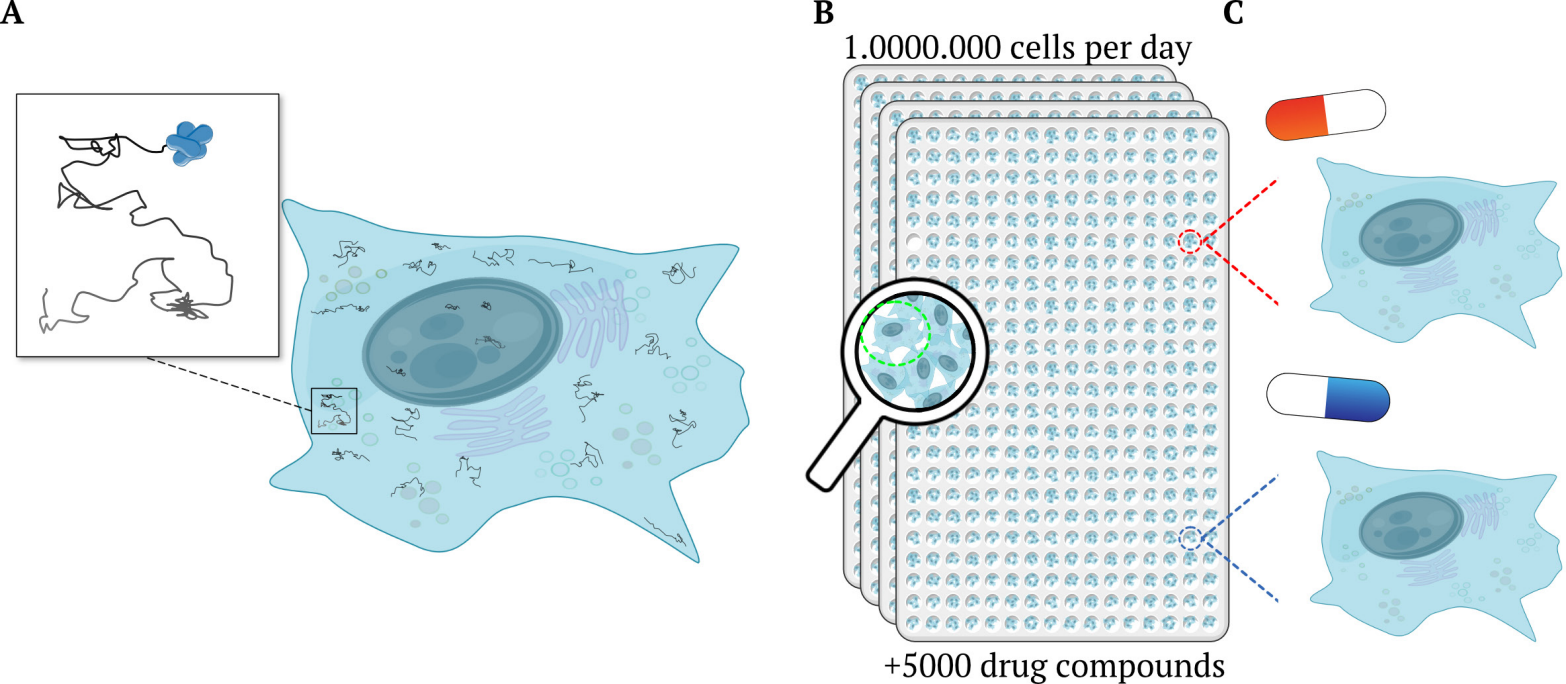
High-throughput single-molecule tracking of proteins across millions of cells. (**A**) Schematic illustration of a cell (blue shape) as taken from a high-throughput single-molecule tracking (htSMT) assay (inset), which tracks the motion of multiple proteins within individual cells (squiggly lines). (**B**) The platform can record the movement of thousands of individual, heterogeneous proteins per cell in over a million cells per day. It does this by automatically collecting a series of images from 384-well plates mounted on a microscope. Each well contains multiples cells and constitutes an independent experiment, enabling researchers to investigate different cell types, and test the effects of various drugs and other molecules and/ or proteins. (**C**) The htSMT results from cells treated with different drugs (indicated as a red or blue pill) can then be used to assess which treatment is likely to work best.

Image credit: Jacob Kæstel-Hansen and Nikos S. Hatzakis. The figure was – with large modifications – generated with elements from Servier Medical Art and Scidraw.io (10.5281/zenodo.3926549; CC BY 4.0).

Overall, the htSMT platform paves the way for a new era in cellular biology and pharmacology, enabling large-scale, automated observations of how proteins move and interact across millions of cells within 24 hours – a feat that until recently remained in the realm of fantasy. This profound increase in scale, together with advanced analytic tools (Muñoz-Gil et al., 2021; Pinholt et al., 2021), promises to unlock even more unresolved information about complex biological pathways, such as those associated with the estrogen receptor. Adapting htSMT to other proteins and cell systems could help construct unique libraries that ultimately link movement to function. Exploiting the full potential of htSMT will further our understanding of the intricate processes occurring within cells and how protein motion contributes to – and depends on – cellular function. Ultimately this could help researchers design new pharmaceutical treatments for controlling certain diseases.
